# Lower Subscapular Nerve Hydrodissection and Subscapularis Re-education for Residual Anterior Shoulder Pain After Superior Labrum Anterior to Posterior Debridement: A Case Report

**DOI:** 10.7759/cureus.93985

**Published:** 2025-10-06

**Authors:** Daichi Naoi, Masashi Kawabata, Daiki Watanabe, Kazuma Miyatake

**Affiliations:** 1 Rehabilitation Center, Sagamihara Kyodo Hospital, Sagamihara, JPN; 2 Rehabilitation, Kitasato University School of Allied Health Sciences, Sagamihara, JPN; 3 Orthopaedic Surgery, Yokohama Minami Kyosai Hospital, Yokohama, JPN; 4 Orthopaedic Surgery, Yokohama City University, Yokohama, JPN

**Keywords:** return to sport, shoulder pain, slap lesion, subscapularis, superior labrum anterior to posterior (slap) lesions, ultrasound-guided hydrodissection

## Abstract

Superior labrum anterior to posterior (SLAP) lesions are frequently identified on magnetic resonance imaging (MRI), particularly in middle-aged patients, yet many remain incidental and asymptomatic. Arthroscopic debridement of type I lesions may provide symptomatic relief; however, unsatisfactory outcomes may occur in some patients. Residual anterior shoulder pain may not be fully attributed to structural pathology alone, indicating a potential contribution of functional deficits, such as dynamic anterior instability and subscapularis dysfunction.

We report the case of a right-handed male amateur arm wrestler in his 40s, who presented with residual anterior shoulder pain (Numerical Rating Scale (NRS) score, 7) and internal rotation weakness (Manual Muscle Testing (MMT) grade, 3) following arthroscopic debridement of a type I SLAP lesion. Steroid injections and hydrodissection of the suprascapular and axillary nerves yielded limited benefits. Targeted ultrasound-guided hydrodissection of the lower subscapular nerve (LSN) was subsequently performed in combination with weekly physiotherapy, including ultrasound-guided subscapularis activation and manual neural mobilization. Pain improved immediately (NRS 3) and resolved within one month (NRS 0), with restoration of internal rotational strength (MMT 5). He resumed competitive arm wrestling four months postoperatively and subsequently won a local tournament.

Residual anterior shoulder pain after SLAP debridement may primarily reflect functional impairment, particularly subscapularis dysfunction, rather than residual labral pathology. Ultrasound-guided lower subscapular nerve (LSN) hydrodissection combined with subscapularis-specific rehabilitation is a promising therapeutic option for refractory postoperative anterior shoulder pain.

## Introduction

Superior labrum anterior to posterior (SLAP) lesions were initially described in 1990 [1] and have since been recognized as a frequent cause of shoulder pain. SLAP lesions are frequently identified on magnetic resonance imaging (MRI), particularly in middle-aged patients, although many of these findings are incidental and asymptomatic [2].

Although arthroscopic debridement of type I SLAP lesions is a viable treatment option, some patients continue to experience residual anterior shoulder pain and have suboptimal outcomes [3]. Moreover, procedures such as arthroscopic synovectomy may be performed as the primary surgical indication, with SLAP debridement performed only when a lesion is incidentally encountered during surgery. These considerations suggest that structural pathology alone cannot fully account for the postoperative symptoms.

Increasing evidence suggests that functional impairments, including dynamic anterior instability, neural mobility restriction, and arthrogenic muscle inhibition, play a central role in refractory anterior shoulder pain [4]. Among these, the subscapularis muscle is significant, serving as the primary dynamic stabilizer of the anterior glenohumeral joint and playing a crucial role in maintaining humeral head centering [5]. Subscapularis dysfunction has been linked to persistent pain and poor return-to-sport outcomes [6].

We report the case of a patient with residual anterior shoulder pain following arthroscopic synovectomy with additional debridement of a type I SLAP lesion that was successfully managed with ultrasound-guided hydrodissection of the lower subscapular nerve (LSN) combined with ultrasound-guided subscapularis re-education.

## Case presentation

Patient information 

A right-handed male amateur arm wrestler in his 40s presented with residual anterior shoulder pain. He had no relevant medical or neurological history. One month after the injury, the patient developed right anterior shoulder pain that persisted despite two months of conservative management, including physical therapy and exercise-based interventions, prompting a referral to our institution.

At the initial visit, the patient’s chief complaint was anterior shoulder pain during flexion with internal rotation, with a Numerical Rating Scale (NRS) score of 7 (0 = no pain and 10 = worst imaginable pain) [7,8]. Physical examination revealed positive Hawkins, Speed, and O’Brien tests. Subscapularis function was assessed using the belly press, bear hug, and lift-off tests, all of which yielded positive results. The range of motion was measured as follows: flexion, 175°; abduction, 170°; external rotation, 70°; and internal rotation (hand-behind-back motion), limited to the T12 vertebral level. Muscle strength was assessed using Manual Muscle Testing (MMT), graded from 0 to 5, with grade 5 indicating normal strength [9]; the results were abduction 5, external rotation 5, and internal rotation 3.

**Figure 1:**
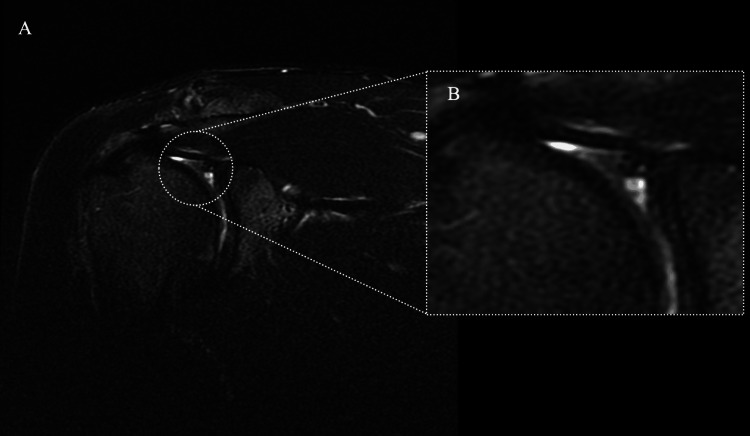
Preoperative T2 fat-suppressed magnetic resonance imaging (MRI) of the right shoulder. Preoperative MRI showing a type I superior labrum anterior to posterior (SLAP) lesion. (A) Coronal view of the right shoulder; (B) Magnified view of the lesion in the superior labrum.

MRI revealed labral injury, articular-sided partial-thickness rotator cuff tears, and bone contusions (Figure 1).

Operative findings and postoperative care

The patient underwent an arthroscopic synovectomy as the primary procedure. Intraoperatively, a type I SLAP lesion was identified with mild detachment at the long head of the biceps anchor, and debridement was performed instead of repair. A posterior labral tear was also observed but was deemed stable, requiring no further intervention. Postoperatively, no range of motion restrictions were imposed, although sling use was permitted until pain subsided.

Timeline and therapeutic interventions

One month postoperatively, anterior shoulder pain persisted during elevation with internal rotation (NRS 5). Ultrasound-guided steroid injections were administered into the rotator cuff interval and the long head of the biceps tendon. Hydrodissection was also performed for the suprascapular and axillary nerves; however, symptomatic relief was minimal. Residual weakness of the subscapularis (MMT 3) suggested functional insufficiency, contributing to impaired dynamic stabilization.

To address this deficit, ultrasound-guided hydrodissection was performed, targeting the LSN between the teres major and latissimus dorsi (Videos 1, 2), in conjunction with weekly physiotherapy. Although this region may also include the thoracodorsal nerve, the LSN was identified by its continuity into the subscapularis, consistent with recent high-resolution ultrasound and anatomical studies [10-12]. Hydrodissection was performed under aseptic conditions using a short-axis, in-plane approach with a 23-gauge needle, using 8 mL of 0.1% lidocaine diluted in saline. Immediate improvements in anterior shoulder pain and internal rotation strength were observed, with no adverse events during or after the procedure.

**Video 1:** Ultrasound-guided hydrodissection targeting the LSN Hydrodissection was performed to target LSN. Although the thoracodorsal nerve may also be visualized in this region, the LSN was identified based on its continuity with the subscapularis. LD: latissimus dorsi muscle; TMa: teres major muscle; SAM: serratus anterior muscle; SSC: subscapularis muscle; LSN: lower subscapular nerve; TN: thoracodorsal nerve

**Video 2:** Ultrasound-guided nerve mobilization for the LSN (short-axis mobilization) Under ultrasound guidance, the LSN (arrowhead) is visualized between the teres major and the latissimus dorsi over the subscapularis. Although the thoracodorsal nerve may also be encountered in this region, the LSN was identified based on its continuity with the subscapularis. Mobilization was facilitated by observing the relative gliding of the teres major and latissimus dorsi over the subscapularis muscle. LSN: lower subscapular nerve

Subsequently, subscapularis activation was reinforced in the belly press, bear hug, and lift-off positions using real-time ultrasound visual feedback to ensure selective contraction and prevent translation of the anterior humeral head. The patient was instructed to perform three sets of 10 repetitions with a five-second hold under manual resistance, discontinuing the exercise if pain worsened (NRS ≥ 2) or compensatory motion appeared. The return-to-play criteria included pain resolution (NRS 0), full recovery of muscle strength (MMT 5), absence of compensatory movement, and the ability to perform sport-specific motions at full intensity.

Results 

Immediately after hydrodissection, the patient’s anterior shoulder pain during elevation and internal rotation decreased from NRS 7 to 3, accompanied by an improvement in internal rotation strength from MMT 3 to 4.

**Figure 2:**
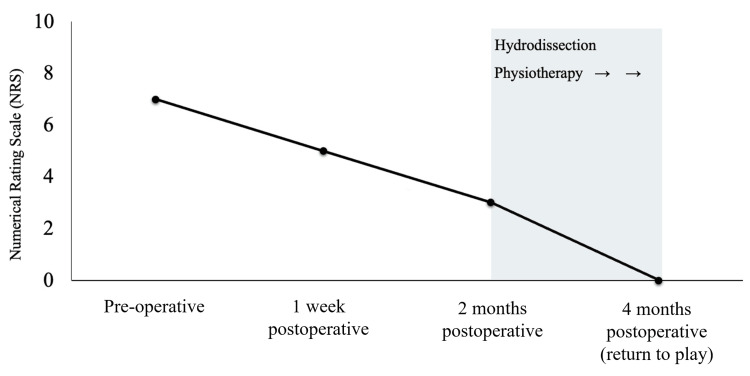
Pain score timeline (Numerical Rating Scale score (NRS) improvement) The mean preoperative NRS score was 7. Although arthroscopic surgery provided temporary improvement, anterior shoulder pain persisted. Seven weeks postoperatively, ultrasound-guided hydrodissection of the lower subscapular and thoracodorsal nerves was performed, resulting in further pain reduction. The pain had completely resolved within one month after the procedure (approximately two months postoperatively), and the patient was able to return to competition after four months.

One month later, the anterior shoulder pain had completely resolved (NRS 0), the Hawkins and bear hug tests were negative, and internal rotation strength had recovered to MMT 5 (Figure 2). Internal rotation (hand-behind-back motion) improved from the T12 to the T7 vertebral levels. Dynamic ultrasound evaluation confirmed improved subscapularis contraction and tendon loading (Videos 3, 4). Before the intervention, the subscapularis contraction was insufficient, with poor force transmission (Video 3A); following the intervention, contraction and tendon tension were clearly observed (Video 3B). Similarly, compensatory activation of the teres major was predominant before intervention (Video 4A) but was reduced after therapy, with restoration of subscapularis contraction (Video 4B).

**Video 3:** Dynamic ultrasound of the subscapularis muscle in the long-axis view (A) Before physiotherapy intervention, contraction of the subscapularis at the terminal range of internal rotation was insufficient, with tension not transmitted to the tendon over the glenoid cavity. (B) Following physiotherapy, subscapularis contraction was evident at the terminal range of internal rotation, with tension transmitted to and exerted on the tendon over the glenoid cavity. Del: deltoid muscle; SSC: subscapularis muscle; CB: coracobrachialis muscle; SHBB: short head of the biceps brachii; H: humerus; GC: glenoid cavity

**Video 4:** Dynamic ultrasound of the subscapularis muscle in the short-axis view (A)  Before the physiotherapy intervention, contraction of the subscapularis was insufficient, with compensatory activation of the teres major being predominant. (B) After physiotherapy, contraction of the subscapularis was evident, whereas compensatory contraction of the teres major appeared to be reduced. LD: latissimus dorsi muscle; TMa: teres major muscle; SAM: serratus anterior muscle; SSC: subscapularis muscle

Physiotherapy was continued weekly for approximately 12-16 weeks, focusing on selective subscapularis activation with real-time ultrasound feedback in the belly press, bear hug, and lift-off positions. These exercises were performed for three sets of 10 repetitions with a five-second hold under manual resistance. The patient resumed competitive arm wrestling four months after surgery and won a local tournament.

## Discussion

The novelty of our patient management lies in the targeting of the LSN, which directly innervates the subscapularis. Few clinical reports have specifically described interventions targeting the LSN despite the subscapularis muscle's central role in anterior glenohumeral stability. 

Previous studies have demonstrated that the debridement or repair of SLAP lesions, especially in middle-aged patients, may lead to unsatisfactory outcomes in patients with residual anterior shoulder pain [3]. These reports support the notion that structural labral pathology alone does not fully account for symptoms and that functional impairments, particularly subscapularis dysfunction, may play a decisive role. Our findings further suggest that targeted hydrodissection of the LSN in conjunction with ultrasound-guided subscapularis re-education directly addresses functional deficits and facilitates rapid recovery.

Hydrodissection is a safe technique to reduce perineural adhesions and restore neural mobility in cases of entrapment neuropathy [13]. However, application of this modality to nerves specifically innervating the dynamic stabilizers of the shoulder has been infrequently reported. Neural mobilization further promotes both peripheral and central adaptations [14], which may have synergistically supported the recovery of this patient.

Importantly, the return to competitive sports after shoulder surgery is often delayed or incomplete, and criteria-based rehabilitation strategies are recommended [15]. In this patient, targeted restoration of subscapularis function facilitated both pain relief and a timely and successful return to arm wrestling competition.

This case highlights the fact that residual anterior shoulder pain after SLAP debridement may reflect functional pathology, particularly subscapularis dysfunction, rather than residual labral abnormalities, highlighting the importance of addressing subscapularis dysfunction as a key factor in postoperative symptoms. Targeted nerve intervention combined with muscle-specific rehabilitation may be a useful therapeutic option for selected patients.

This study is limited by its single-patient design and absence of long-term follow-up. Further prospective studies are required to determine whether subscapularis-targeted interventions consistently improve the outcomes in larger patient populations.

## Conclusions

This case suggests that residual anterior shoulder pain after SLAP debridement may reflect a functional rather than structural pathology. Targeted ultrasound-guided hydrodissection of the LSN combined with subscapularis-specific rehabilitation may be a useful therapeutic option to relieve pain and restore function, potentially allowing a return to sports. These findings highlight the importance of considering subscapularis dysfunction as a possible cause of refractory postoperative shoulder pain.
